# Comparison of the effects of different calorie amounts of enteral nutrition in hypercatabolism associated with ghrelin-POMC in endotoxemic rats

**DOI:** 10.1186/s12986-022-00663-7

**Published:** 2022-04-15

**Authors:** Jianfeng Duan, Minhua Cheng, Yali Xu, Shaoqiu Tang, Xiaoyao Li, Yan Chen, Huimin Lu, Tao Gao, Wenkui Yu

**Affiliations:** 1 Affiliated Drum Tower Hospital, Medical School of Nanjing University, 321st Zhongshan Road, Nanjing, Jiangsu People’s Republic of China; 2 Nanjing University Hospital, 22nd Hankou Road, Nanjing, Jiangsu People’s Republic of China; 3 Medical School, Nanjing University, 22nd Hankou Road, Nanjing, Jiangsu People’s Republic of China

**Keywords:** Endotoxemia, Enteral nutrition, Metabolic regulation, Gastrointestinal hormones, Hypercatabolism, Neuropeptide

## Abstract

**Background:**

Hypercatabolism often occurs in critically ill patients, and it increases infection rates and mortality in these patients. Enteral nutrition (EN) is commonly used in case of hypercatabolism. However, the effect of amount of calories in EN on hypercatabolism remains unexplored.

**Objective:**

Here, we compared the effect of low-calorie, medium-calorie and high-calorie EN on hypercatabolism in the acute phase of endotoxemia, which is associated with gastrointestinal hormones and hypothalamic neuropeptide proopiomelanocortin (POMC).

**Methods:**

Overall 84 adult male Sprague–Dawley rats were used for research. A set of rats were divided into 5 groups, Control (NS) and lipopolysaccharide (LPS) groups were fed a standard chow diet; LPS + L (LPS + 40 kcal/kg/day EN), LPS + M (LPS + 80 kcal/kg/day EN) and LPS + H (LPS + 120 kcal/kg/day EN) groups received EN through a gastric tube for 3 days. Another set of rats were used for parallel control experiment and divided into 5 groups: NS + F (saline + fasting) and LPS + F (LPS + fasting) groups were given no food, NS + L (saline + 40 kcal/kg/day EN), NS + M (saline + 80 kcal/kg/day EN) and NS + H (saline + 120 kcal/kg/day EN) groups received EN through a gastric tube for 3 days. Hypercatabolism was evaluated by assessing skeletal muscle protein synthesis and atrophy, insulin resistance, and corticosterone levels. Moreover, serum inflammatory factors, gastrointestinal hormones, hypothalamic ghrelin, growth hormone secretagogue receptor-1α, hypothalamic neuropeptide, and intestinal injury indicators were detected.

**Results:**

Low-calorie EN effectively increased serum and hypothalamic ghrelin possibly due to slight intestinal barrier damage, thereby decreasing hypothalamic POMC expression; consequently, it alleviated rat insulin resistance, reduced blood cortisol levels and muscle atrophy, and improved the survival rate of rats in the acute phase of endotoxemia. Interestingly, with an increase in calories in enteral nutrition, the aforementioned effects did not increase.

**Conclusions:**

Low-calorie EN could effectively increase gastrointestinal hormone ghrelin by reducing intestinal damage and suppressing POMC expression to ameliorate hypercatabolism when compared with medium-calorie and high-calorie EN. Therefore Low-calorie EN may be preferred for providing EN in the acute stage of endotoxemia.

**Supplementary Information:**

The online version contains supplementary material available at 10.1186/s12986-022-00663-7.

## Introduction

Nutrition therapy plays an important role in intensive care unit (ICU) [1, 2]. Various randomised controlled trials and systematic reviews have reported that early enteral nutrition (EN) is associated with better outcomes [3, 4]. However, the optimum amount of calories to be provided to critically ill patients though EN has not been reported to date, especially for patients in the acute stage of endotoxemia who have severe hypercatabolism and nutritional irresponsiveness; moreover, the available evidence is conflicting and no consensus has been reached on this issue so far.

Studies have shown that when isocaloric nutrition is provided to patients, their short-term mortality is improved significantly, whereas under- or over-nutrition negatively affects the prognosis of patients [5–9]. In contrast, some data suggest that hypocaloric feeding (40 to 60% of calculated caloric requirements) and full feeding (70 to 100%) exhibit no difference in terms of mortality, risk of pneumonia, or duration of mechanical ventilation, and the incidences of bloodstream infections and renal replacement events decreased when fewer calories were administered [10–20]. Moreover, some studies have indicated that low-calorie feeding (15–25%) could shorten the duration of mechanical ventilation and improve overall mortality [19, 21]. Although the administered calories should be in line with the dissipated energy, the optimal calorie at the corresponding time may vary across patients and has not been studied in detail to date.

Parenteral nutrition and enteral nutrition do not seem to differ in terms of nutritional support alone. However, currently, EN is preferred because EN is not only a nutrition support, but also a therapeutic that provides intestinal protection and metabolic regulation [1, 2, 22, 23]. Several studies have confirmed that EN provides nutrients such as calories and proteins, and plays a role in regulating the immune and metabolic state though the gastrointestinal mucosal barrier, intestinal endocrine and immune systems, and normal flora colonised in the intestine, which is extremely important to human body, and can effectively reduce the damage to the body caused by systemic stress in critically ill patients [24–27]. Trophic feeding can confer protection to the intestinal barrier [28], however,the optimal amount of calories in EN required for the metabolic regulation has not been elucidated to date.

In our previous studies, we found that central regulation plays an important role in hypercatabolism of critically ill patients and confirmed that hypothalamic POMC is one of the key regulatory factors for hyporesponsiveness to nutrition [29–31]. LPS injection caused a rapid rise in proinflammatory cytokines such as IL-6 and TNF -α within 1 to 3 h, peaked at 4 to 6 h, and then decreased slightly and remained at high levels [32, 33].Meanwhile, LPS injection led to significant increase in hypothalamic inflammation and expression of proopiomelanocortin (POMC) as well as muscle wasting [31]. Ghrelin expression level increased briefly and then decreased significantly in sepsis [34, 35]. Besides, gastric feeding could activate hypothalamic AMPK-autophagy and suppress POMC expression though gastrointestinal hormones, mainly ghrelin, to ameliorate hypercatabolism when compared with jejunal feeding [36].

The main purpose of this study was to compare the effects of different calories EN on regulation of hypercatabolism in endotoxemic rats, and to determine the optimum amount of calorie in the acute phase of endotoxemia.

## Materials and methods

### Animals

In total 84 adult male Sprague–Dawley rats (weighing 276.97 ± 12.63 g, 8–10 weeks) were obtained from the Animal Research Center of Nanjing Medical University. The animals were housed under regular lighting conditions (12 h:12 h dark/light cycle) in a constant temperature environment with free access to tap water and standard rat pellet chow. This study was performed in accordance with the Institutional Animal Care and Use Committee of Nanjing University.

### EN through gastric tube and model of endotoxemia

The model of endotoxemia was induced, and EN was provided through a gastric tube, as described previously. Briefly: a puncture through the gastric corpus was made using a thick needle (14G, 2.0 mm OD); 2.0 cm of the silicon rubber tube (medical grade, 0.025 inch ID; 0.029 inch OD, Helix Medical, USA) was inserted distally into the gastric corpus, and a purse-string suture was made in the gastric corpus. The catheter was subcutaneously tunnelled to the shoulder region and exited at the midpoint of the cervicodorsal region and passed through a tightly coiled stainless steel spring, and connected through a freely rotating swivel to a 50 mL syringe pump. Further, all rats received 0.9% saline at a rate of 1 mL/h through the tube for 24 h for surgical recovery. After EN access was established, the rats received an intraperitoneal injection of lipopolysaccharide (LPS; 10 mg/kg, Escherichia coli serotype 055:B5, Sigma) or saline.

### Experimental protocol

A set of rats (Experiment 1, n = 54) were randomly divided into 5 groups: NS (control group, n = 6), LPS (n = 12), LPS + L (LPS + 40 kcal/kg/day, n = 12), LPS + M (LPS + 80 kcal/kg/day, n = 12) and LPS + H (LPS + 120 kcal/kg/day, n = 12). Sham surgery was performed in the NS and LPS groups. Then NS and LPS groups were fed standard food ad libitum and were given the same volume of saline as LPS + L group through a gastric tube. LPS + L, LPS + M and LPS + H groups received EN solutions at 40(about 20% of calculated caloric requirements), 80 and 120 kcal/kg/day (Ensure Enteral Nutrition powder was diluted with saline at 27.9 g/100 mL, 1.0 kcal/mL) respectively through a gastric tube. Another set of rats (Experiment 2, n = 30) were used for parallel control experiment to decipher the feeding effects and divided into 5 groups: NS + F (saline + fasting) and LPS + F (LPS + fasting) groups were given no food but were given the same volume of saline as LEN groups through a gastric tube for three days, NS + L (saline + 40 kcal/kg/day EN), NS + M (saline + 80 kcal/kg/day EN) and NS + H (saline + 120 kcal/kg/day EN) groups received EN through a gastric tube for 3 days (subgroups and sample size seen in Additional file 1: Chart S1).

Nutrition powder was purchased from ABBOTTLABORATORIES B.V. containing 14.1% protein, 54.2% carbohydrates, 31.7% lipids, electrolytes, and multivitamins, with a nonprotein energy and nitrogen ratio of 153:1 (640 kJ/g nitrogen). The standard food was purchased from XIETONG Biotics Inc. JIANGSU province, containing 18% protein, 58% carbohydrates, 24% lipids, electrolytes, and multivitamins. The energy density of the food was about 3.5kccal/g.

After 3 days of nutrition support,all rats were fasted overnight (12 h), and the body weight (BW) change was measured; and then, the rats were sacrificed with an overdose of phenobarbital sodium. Serum samples were collected to measure inflammatory factors, corticosterone and gastrointestinal hormones and fasting blood glucose and insulin levels. The hypothalamic tissue, extensor digitorum longus (EDL), and ileum were obtained and washed in pre-cooled saline from each rat and kept at -80 °C until analysis.

### Western blotting

Animal tissues were homogenised and incubated for 60 min at 4℃ in lysis buffer and separated through SDS/PAGE for Western blot analyses. Primary antibodies included: Ghrelin (Abcam #ab129383), growth hormone secretagogue receptor-1α (GHS-R1α; Abcam #ab95250), protein kinase B (Akt; CST #4691), p-Akt (CST #4060), mammalian target of rapamycin (mTOR; CST #2983), p-mTOR (CST #5536), muscle ring-finger1 (MURF-1; Abcam #ab172479), muscle atrophy F-box (MAFBx; Abcam #ab168372), MUC2(Abcam #ab272692), ZO-1(WUHAN SANYING #21773-1-AP),GAPDH(Servicebio #GB12002) and β-actin (Servicebio #GB12001).

### Measuremnt of muscle atrophic gene, hypothalamic neuropeptides gene and intestine inflammation cytokines gene

Real-time PCR was used to detect gene expression. The total RNA was isolated from hypothalamus and extensor digitorum longus muscle using Trizol reagent (Invitrogen, USA) according to the manufacturer’s instructions. Gene expression was analysed using the Rotor-Gene Real-Time Analysis Software 6.1. Glyceraldehyde phosphate dehydrogenase (GAPDH) was used as an internal control gene to normalize the target mRNAs of musle and intestine, β-actin was used as an internal control gene to normalize the target mRNAs in hypothalamic. and gene expression was compared among groups using the ΔΔCT method. The primer sequences are listed in Table 1.

**Table 1:** All primers for RT-PCR assay

Gene		Primers
MuRF1	Forward	5′-CCAGGTGAAGGAGGAACT-3′
	Reverse	5′-TTGGCACTCAAGAGGAAGG-3′
MAFbx	Forward	5′-CTTGTGCGATGTTACCCA-3′
	Reverse	5′-GTGAAAGTGAGACGGAGC-3′
POMC	Forward	5′-CCTCCTGCTTCAGACCTCCA-3′
	Reverse	5′-GGCTGTTCATCTCCGTTGC-3′
AgRP	Forward	5′-TGAAGGGCATCAGAAGGT-3′
	Reverse	5′-CACAGGTCGCAGCAAGGT-3′
CART	Forward	5′-GAAGTACGGCCAAGTCCCCA-3′
	Reverse	5′-AAGAAGTTCCTCGGGGACAGT-3′
NPY	Forward	5′-GTGTTTGGGCATTCTGGCTG-3′
	Reverse	5′-AGTGTCTCAGGGCTGGATCT-3′
ghrelin	Forward	5′-TGGTGTCTTCAGCGACTATCTGC-3′
	Reverse	5′-TCTCTGCTGGGCTTTCTGGTG-3′
GHS-R1α	Forward	5′-GTGTCCAGCGTCTTCTTCTTTC-3′
	Reverse	5′-GTGTCCAGCGTCTTCTTCTTTC-3′
MUC2	Forward	5′-GCTGAACACTGGTGCTCCCTAC-3′
	Reverse	5′-GTCCTCGTTGTTCTGACAGTTGC-3′
ZO-1	Forward	5′-CGGGCTACCTTATTGAATGTCC-3′
	Reverse	5′-GAGCGAACTGAATGGTCTGATG-3′
β-actin	Forward	5′-TGCTATGTTGCCCTAGACTTCG-3′
	Reverse	5′-GTTGGCATAGAGGTCTTTACGG-3′
GAPDH	Forward	5′-CTGGAGAAACCTGCCAAGTATG-3′
	Reverse	5′-GGTGGAAGAATGGGAGTTGCT-3′

### Gastrointestinal hormone measurements

Total ghrelin, acylated ghrelin, desacylated ghrelin, PYY, and GLP-1 in serum were measured using total ghrelin enzyme-linked immunosorbent assay (ELISA) kit (F3639-A, Fankew), acylated ghrelin ELISA kit (F40298-A, Fankew), desacylated ghrelin ELISA kit (F40300-A, Fankew), PYY ELISA kit (F3521-A, Fankew), and GLP1 ELISA kit (F2909-A, Fankew), respectively, according to the manufacturer’s instructions.

### Measurements of fasting blood glucose, insulin, leptin, corticosterone, and inflammation cytokines

Blood glucose was measured using an automatic blood glucose metre (Roche). Serum insulin and leptin were measured using ELISA (F3462-A,F3449-A,Fankew). Homeostatic model assessment for insulin resistance index was performed according to the following formula: [fasting glucose levels (mmol/L)] × [fasting serum insulin (mIU/mL)]/ 22.5. Serum corticosterone was measured by RIA (MP Biomedicals) according to the manufacturer’s instructions. Inflammatory cytokines IL-1 and TNF-α in the serum samples were measured by using ELISA (F2923-A, F3056-A, respectively, Fankew).

### Measurement of inflammation cytokines and injury indicators in the ileum

Inflammatory cytokines IL-1 and TNF-α in the ileum were measured using ELISA (JER-051, and JER-06, respectively Joyeebio). The procedure was described as follows: appropriate amount of tissue was taken and cleaned in pre-cooled PBS (0.02 mol/L, pH 7.0–7.2), and homogenized on the ice. The prepared homogenate was centrifuged at 5000 × g for 5 min, and the supernatant was retained for ELISA. ZO-1 and MUC2 were measured using Werstern Blot.

### Statistical analyses

The experimental data are expressed as means ± standard error (SE). Statistical analyses were performed using the SPSS for Windows (version 26.0; SPSS Inc., Chicago, IL). Differences among the groups were assessed by ANOVA followed by the post-hoc least significance difference test. A *p* values < 0.05 was considered significant.

## Results

### Effects of different calories of enteral nutrition on survival rate and body weight in endotoxemia rat

**Fig. 1:**
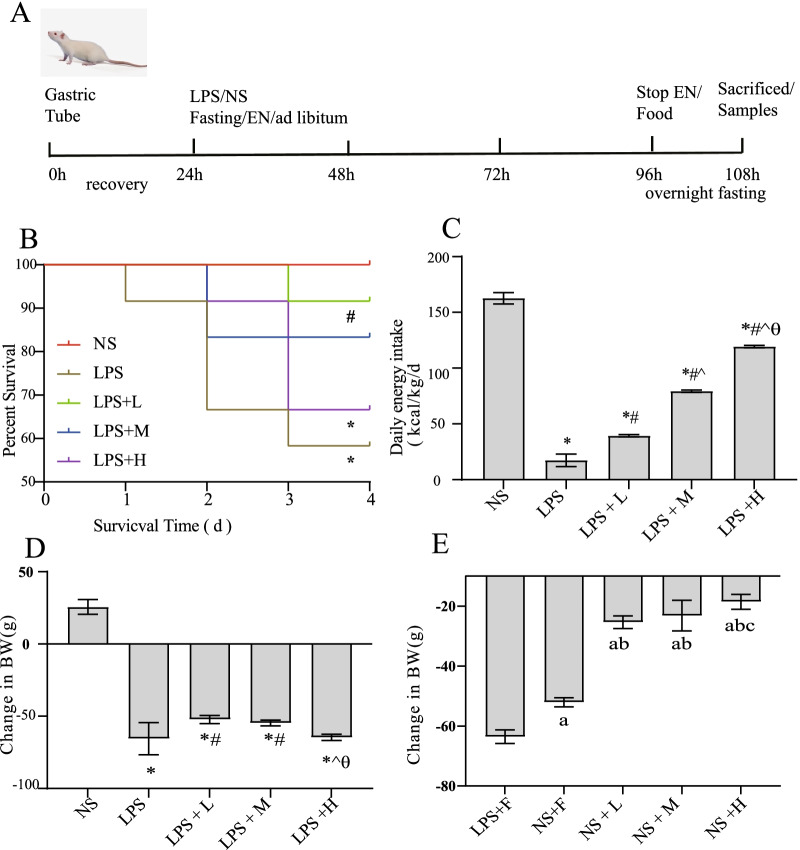
Effects of EN with different calories on survival and BW during endotoxemia. **A** Schematic timeline of the experimental procedure; **B** survival rate; **C** daily calorie intake; **D**, **E** BW; *P < 0.05 compared with NS group; #*P* < 0.05 compared with LPS group; ^*P* < 0.05 compared with LPS + L group; θP < 0.05 compared with LPS + M group. aP < 0.05 compared with LPS + F group; bP < 0.05 compared with NS + F group; cP < 0.05 compared with NS + L group

As shown in Fig. 1, LPS injection resulted in a considerable decrease in the BW, survival rate and food intake. The survival rate was significantly improved in LPS + L group, whereas it was only slightly affected in the LPS + M and LPS + H groups. Interestingly, weight loss did not decrease with an increase in calories, and was significantly ameliorated in the LPS + L and LPS + M groups. However, in the rats with saline injected, weight loss decreased with an increase in calories.

### The effect of different calories of EN on inflammatory factors in serum in the endotoxemic rats

**Fig. 2:**
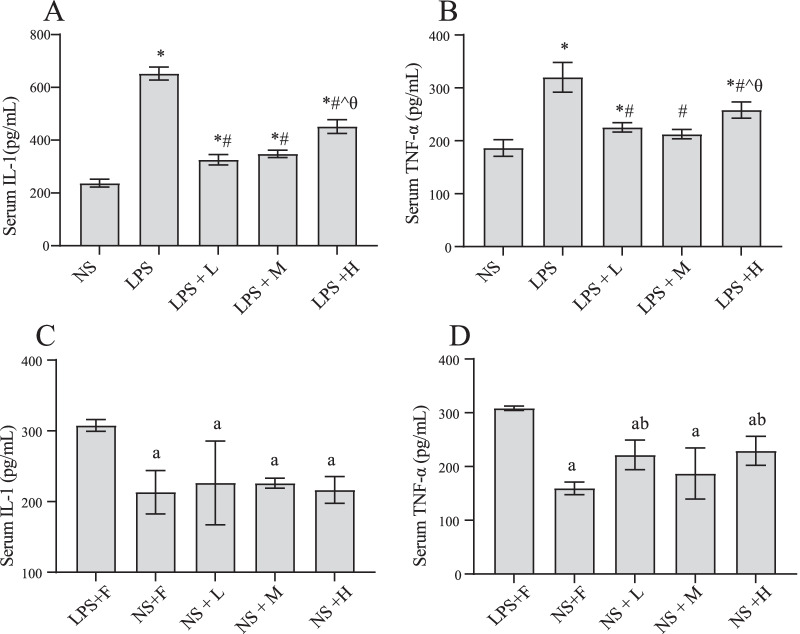
Effects of EN with different calories on inflammatory cytokine levels during endotoxemia. **A**, **C** serum IL-1, **B**, **D** TNF-a. ^*^P < 0.05 compared with NS group; ^#^*P* < 0.05 compared with LPS group; ^*P* < 0.05 compared with LPS + L group; ^θ^*P* < 0.05 compared with LPS + M group. ^a^*P* < 0.05 compared with LPS + F group; ^b^*P* < 0.05 compared with NS + F group

As shown in Fig. 2, LPS injection led to a significant increase in the levels of serum inflammatory factors IL-1 and TNF-α. After the intervention of EN, the levels of serum inflammatory factors decreased significantly. However, no significant differences were observed in serum IL-1 and TNF-α levels between the LPS + L and LPS + M groups, and the IL-1 and TNF-α levels in the LPS + H group were significantly higher than those in the LPS + L and LPS + M groups respectively. However, in the rats with saline injected, no notable differences were observed in serum IL-1 and TNF-α levels among three nutrition-treated groups.

### The effect of different calories of EN on blood glucose, leptin, and corticosterone levels in the endotoxemic rats

**Fig. 3:**
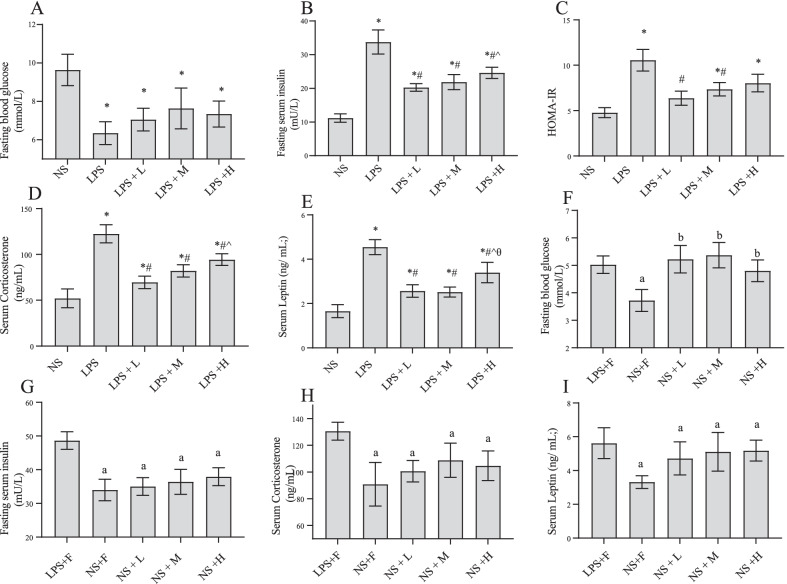
Effects of EN with different calories on **A**, **F** fasting blood glucose; **B**, **G** fasting serum insulin; **C** HOMA-IR; **D**, **H **corticosterone; **E**, **I** leptin during endotoxemia. ^*^P < 0.05 compared with NS group; ^#^*P* < 0.05 compared with LPS group; ^*P* < 0.05 compared with LPS + L group; ^θ^*P* < 0.05 compared with LPS + M group. ^a^*P* < 0.05 compared with LPS + F group

As shown in Fig. 3, LPS injection resulted in severe hypoglycaemia, insulin resistance, and increased levels of leptin and corticosterone. In the LPS + L and LPS + M groups, leptin and corticosterone levels and insulin resistance significantly reduced, whereas the LPS + H group exhibited slight effect on insulin resistance compared with the LPS group. The extent of insulin resistance and corticosterone levels in the LPS + M group appeared higher than those in the LPS + L, although the difference were nonsignificant. Under fasting conditions, LPS injection resulted in higher level of glucose than saline injection. There was no notable differences in blood glucose, leptin, and corticosterone levels among three nutrition-treated groups in saline-injected rats.

### The effect of different calories of EN on gastrointestinal hormones in endotoxemia rats

**Fig. 4:**
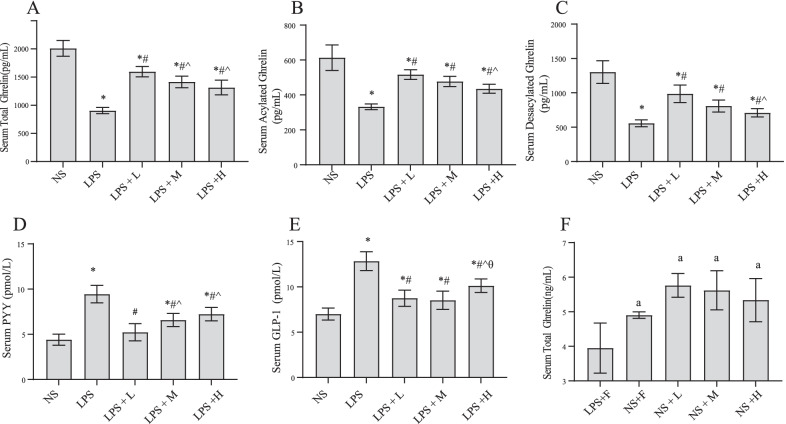
Effects of EN with different calories on **A**, **F** serum total ghrelin; **B** serum acylated ghrelin; **C** serum desacylted ghrelin; **D** serum PYY; **E** serum GLP-1. ^*^P < 0.05 compared with NS group; ^#^*P* < 0.05 compared with LPS group; ^*P* < 0.05 compared with LPS + L group; ^θ^*P* < 0.05 compared with LPS + M group. ^a^*P* < 0.05 compared with LPS + F group

We measured the levels of gastrointestinal hormones in the rats. As shown in Fig. 4. LPS injection resulted in a significant decrease in total, acylated and deacetylated ghrelin in serum, and an increase in serum GLP-1 and PYY. After EN, serum ghrelin levels ameliorated significantly, and serum CLP-1 and PYY levels were significantly reduced. Among the three EN groups, the serum total ghrelin, acylated ghrelin and desacylated ghrelin decreased but the PYY levels increased with an increase in the EN calories. The levels of serum ghrelin, acylated ghrelin and desacylated ghrelin in the LPS + L group were higher than those in the LPS + H groups, however, the difference in acylated and desacylated ghrelin between the LPS + L and LPS + M groups was nonsignificant. In rats with saline injected, there was no notable differences in total ghrelin among three nutrition treated groups.

### The effect of different calories of EN on hypothalamic ghrelin and GHS-R1α levels in the endotoxemic rats

**Fig. 5:**
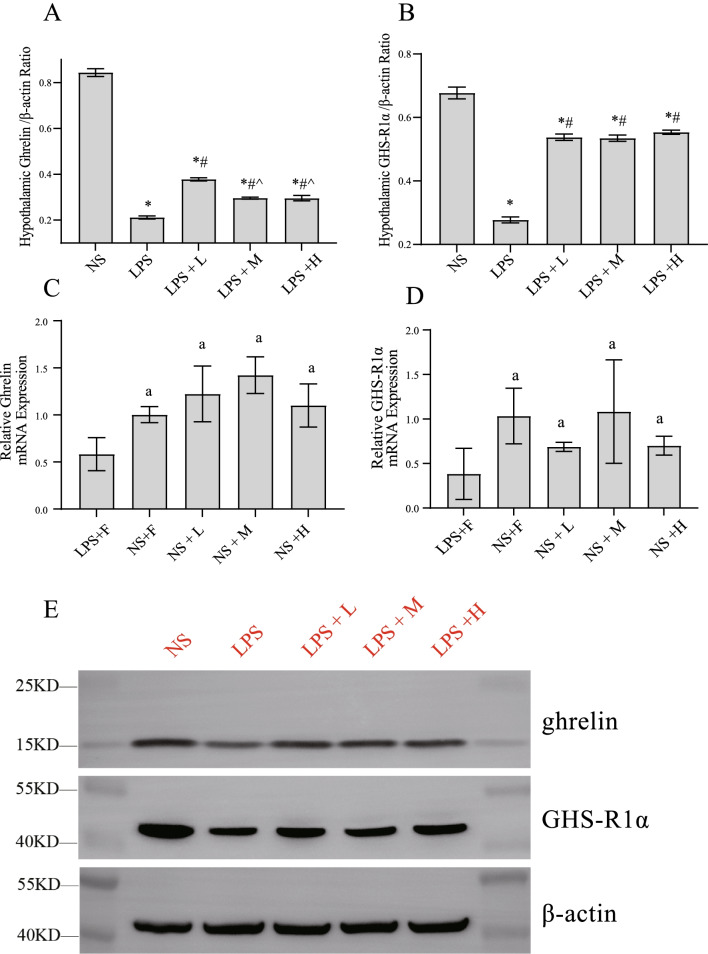
Effects of EN with different calories on hypothalamic ghrelin and GHS-R1α during endotoxemia. **A**, **C** hypothalamic ghrelin; **B**, **D** GHS-R1α; **E** Western blotting of hypothalamic ghrelin and GHS-R1α, β-actin wasused as control. ^*^P < 0.05 compared with NS group; ^#^*P* < 0.05 compared with LPS group; ^*P* < 0.05 compared with LPS + L group; ^a^*P* < 0.05 compared with LPS + F group; ^b^*P* < 0.05 compared with NS + F group; ^c^*P* < 0.05 compared with NS + L group

We measured the hypothalamic ghrelin and GHS-R1α levels and assessed the expression of hypothalamic neuropeptide in the rats. As shown in Fig. 5, LPS injection reduced the levels of ghrelin and GHS-R1α in the hypothalamus. These changes were significantly alleviated after providing EN. Among the EN groups, the LPS + L group exhibited more obvious effects in terms of increasing hypothalamic ghrelin compared with the LPS + M and LPS + H groups. Although EN ameliorated the expressions of GHSR-1α, no significant difference was observed between the EN groups. In rats injected with normal saline, EN with different calories had no significant effect on ghrelin or GHSR-1α level in hypothalamus.

### The effect of different calories of EN on hypothalamic neuropeptide levels in the endotoxemic rats.

**Fig. 6:**
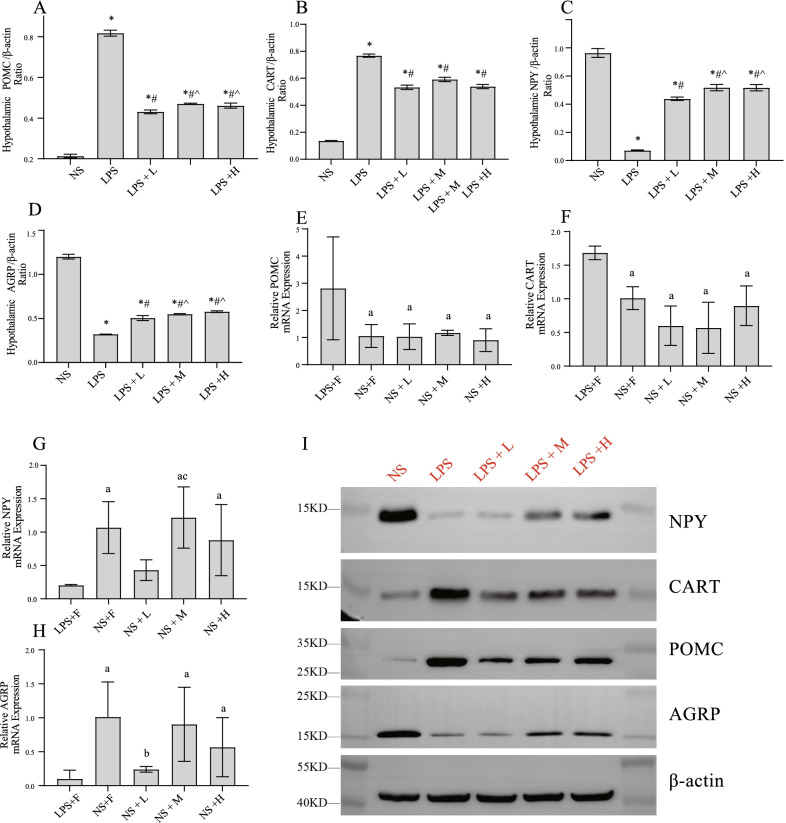
Effects of EN with different calories on hypothalamic neuropeptide a during endotoxemia. The expression of **A**, **E** POMC; **B**, **F** CART; **C**, **G** NPY; **D**, **H** AGRP. β-Actin was used as control. **I** Western blotting of hypothalamic neuropeptide, β-actin wasused as control. ^*^P < 0.05 compared with NS group; ^#^*P* < 0.05 compared with LPS group; ^*P* < 0.05 compared with LPS + L group; ^a^*P* < 0.05 compared with LPS + F group

As shown in Fig. 6, we assessed the expression of hypothalamic neuropeptide in the rats. LPS injection reduced the expressions of orexin AGRP and NPY. Additionally, the expressions of POMC and CART were significantly increased in endotoxemia. In endotoxemia rats, the LPS + L group exhibited more obvious effects in terms of reducing POMC level compared with the LPS + M and LPS + H groups. However, the LPS + M and LPS + H groups exhibited a stronger effect in increasing AGRP and NPY levels. In rats injected with saline, there was no significant difference in hypothalamic neuropeptide levels between enteral nutrition groups with different calories.

### The effect of different calories of EN on muscle protein synthesis and acute muscle atrophy in the endotoxemic rats.

**Fig. 7:**
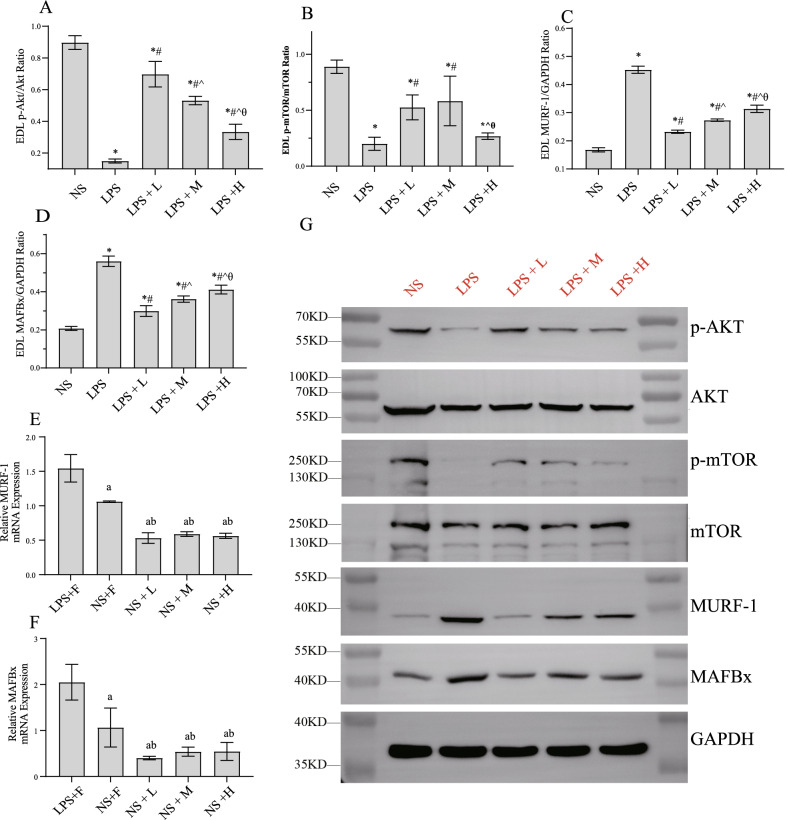
Effects of EN with different calories on muscle protein synthesis and acute muscle atrophy during endotoxemia. The expression of **A** p-Akt/Akt; **B** p-mTOR/mTOR; **C**, **E** MURF-1; **D**, **F** MAFBx. **G** Western blotting of muscle protein synthesis and acute muscle atrophy, GAPDH was used as control. ^*^P < 0.05 compared with NS group; ^#^*P* < 0.05 compared with LPS group; ^*P* < 0.05 compared with LPS + L group; ^a^*P* < 0.05 compared with LPS + F group; ^b^*P* < 0.05 compared with NS + F group

As shown in Fig. 7, LPS injection resulted in a significant decrease in p-Akt and p-mTOR in EDL, which are known to facilitate protein synthesis and inhibit proteolysis in skeletal muscle fibers. In addition, LPS injection led to an increase in MURF-1 and MAFBx levels, which contributed to proteolysis by the proteasome. These results suggested that in this endotoxemia model, muscle protein synthesis was suppressed and atrophy was triggered. EN intervention resulted in a substantial maelioration of p-Akt and p-mTOR. The increased expressions of MURF-1 and MAFBx were significantly reduced. Among the three EN groups, the LPS + L group exhibited a more significant increase in the p-Akt and a more significant decrease in MURF-1 and MAFBx levels compared with the LPS + M group, although no significant difference was observed in the p-mTOR levels between the LPS + L and LPS + M groups. Thus muscle protein synthesis and acute muscle atrophy were highly significantly ameliorated in the LPS + L group compared with those in the LPS + M group. However, the LPS + H group showed the least effect on muscle protein synthesis and atrophy pathway in EDL. In rats with saline injected, the levels of MURF-1 and MAFBx in group NS + L were slightly lower than those in groups NS + M and NS + L, but there was no significant differences.

### The effect of different calories of EN on gastrointestinal barrier function in the endotoxemic rats

**Fig. 8:**
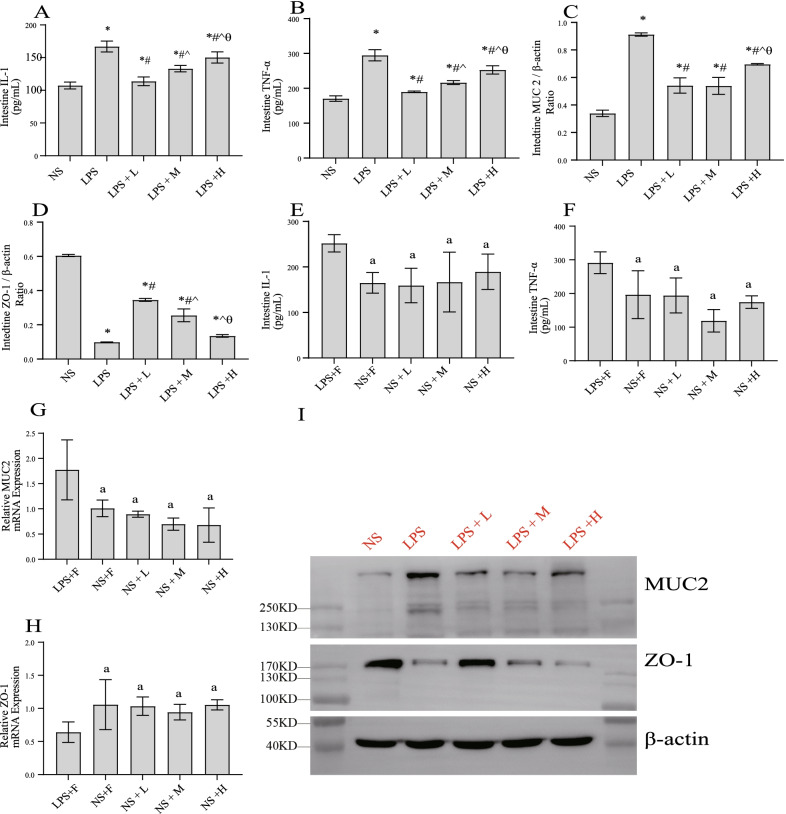
The effect of different calories of enteral nutrition on gastrointestinal barrier function in the endotoxemia rats. The expression of **A**, **E** IL-1, **B**, **F** TNF-α; **C**, **G** MUC2; **D**, **H** ZO-1; **I** Western blotting of ZO-1 and MUC2, β-Actin was used as control. ^*^P < 0.05 compared with NS group; ^#^*P* < 0.05 compared with LPS group; ^^^*P* < 0.05 compared with LPS + L group; ^a^*P* < 0.05 compared with LPS + F group

We further investigated the intestinal damage (Fig. 8). Compared with the NS group, LPS injection caused severe intestinal barrier damage, enhanced intestinal inflammation, decreased ZO-1 expression, and increased MUC2 expression. EN rescued these levels to some extent. In the LPS + L group, the intestinal inflammation and MUC2 levels was notably reduced, and the expression of ZO-1 was significantly recovered. The increase in calories of EN did not enhance the protective effect of EN on the intestinal tract. On the contrary, the levels of IL-1 and TNF-α in the LPS + M group were significantly higher than these in LPS + L, and the LPS + M group exhibited lower expression of ZO-1 than the LPS + L group, however, no significant difference in MUC2 level was observed between the LPS + L and LPS + M groups. The LPS + H group exhibited severe inflammation and intestinal barrier damage, in addition, the ZO-1 expression level in this group was not obviously different from that in the LPS group. In saline injected rats, no remarkable differences were observed in IL-1,TNF-α, MUC2 or ZO-1 among three nutrition treated groups.The above results indicated that LEN can effectively improve the intestinal mucosal mechanical barrier damage in the acute stage of endotoxemia. During this period, excessive EN was not conducive to the protection of the intestinal barrier.

## Discussion

Optimal nutritional therapy, required an individually adapted delivery of calorie as close as possible to patient’s actual energy expenditure (EE), is associated with better clinical outcomes [37, 38]. Indirect calorimetry (IC) is the best tool and recommended clinical standard for measuring EE and monitoring the variations over time of patients, especially for these patients who is on mechanical ventilation, and the guidelines also suggest isocaloric nutrition can be progressively implemented after the early phase of acute illness if indirect calorimetry is used [9]. However, IC has so far been rarely used in clinical practice because of the lack of equipment, the demand for manpower and the high cost. Predictive equations (20–25 kcal/kg/d) is still commonly used in clinical, which is associated with significant inaccuracy (up to 60%) [39, 40]. Therefore the optimum amount of calorie is still unclear, and due to conflicting evidence, no consensus has been reached so far. In addition to providing the energy, EN has therapeutic effects, including nourishment and metabolic regulation. These aspect indicate its superiority to parenteral nutrition. However, currently, only a few studies have discussed its effect on metabolic regulation. Exploring optimal calorie of EN for metabolic regulation is of great significance for clinical treatment.

In this study, different calories were provided through EN to the rats with endotoxemia to compare the effects of the amount of calorie on the metabolic regulation of rats in the acute phase of endotoxemia. The results showed that low-calorie EN can effectively increase serum ghrelin levels in the acute phase, thereby increasing hypothalamic ghrelin levels, reducing POMC expression, alleviating rat insulin resistance, reducing blood cortisol levels, and reducing muscle atrophy to ultimately improve the survival rate of septic rats. Interestingly, with an increase in calories in EN, no changes was observed in the aforementioned effects.

Hypercatabolism occurs early, rapidly, and widely in critically ill patients, and plays a key role in the pathogenesis of post-critical debilitation. The loss of lean body mass leads to an increase in the infection rate and mortality. Skeletal muscle is the main target organ for hypercatabolism [41–43]. In our experiments, compared with the medium-calorie and high-calorie EN, low-calorie EN was found to be more effective in alleviating insulin resistance and reducing the levels of cortisol. The expressions of MURF and MAFBx in EDL was also reduced more obviously in the LPS + L group, suggesting that the rats in this group underwent less muscle wasting. These results suggested that in the acute stage of endotoxemia, EN not only provides energy, but also regulates metabolism. In the acute phase, low-calorie EN may be more conducive to improving the body’s high catabolism.

In our earlier study, we found that hypercatabolism was elicited by the elevated POMC expression during endotoxemia [30, 44]. POMC could be cleaved into α-MSH and ACTH to activate the HPA axis. The binding of α-MSH to its receptor MC4R/MC3R promotes energy expenditure and weight loss, which are the vital determinants of muscle wasting during endotoxemia [45, 46]. AGRP is an antagonist of MC4R [47, 48]. Central injection of AGRP can reduce muscle atrophy and systemic inflammation in rats with chronic kidney disease [49]. In this study, we observed that in the LPS + L group, the elevated expression of POMC was effectively attenuated compared with that in the LPS + M and LPS + H groups, which was consistent with altered hypermetabolism. In addition, in the hypothalamus, the levels of orexigenic NPY and AGRP and the anorexic neuropeptide CART in the LPS + L group were lower than those in the LPS + M and LPS + H groups respectively. This results indicated that EN could effectively alleviate hypercatabolismin the rats in the acute phase of endotoxemia by regulating the expression of POMC/CART and AGRP/NPY in the hypothalamus.

Hypothalamic AMPK-autophagy is a pivotal pathway for the expression of POMC [30, 50]. Ghrelin administration via the peripheral or central pathway could increase intracellular Ca2 + release to initiate the CaMKKβ pathway and promote AMPK phosphorylation in the arcuate nucleus with binding to hypothalamic GHS-R1α [51, 52]. Ghrelin is a multifaceted peptide hormone. In addition to stimulating growth hormone release, it promotes appetite, diminishes fat utilization, increases BW, preserves positive energy balance and modulates energy metabolism [53, 54]. It is effective to protect organs against infections, ischaemia reperfusion and other diseases [55–60]. In our study, the LPS + L group exhibited a substantially higher ghrelin level in serum and hypothalamus compared with the LPS + M and LPS + H groups, and it significantly recoverd hypothalamic GHS-R1α expression compared with the LPS group. This result suggested that in the LPS + L group, the expression of POMC could be suppressed by efficiently increasing ghrelin in serum and hypothalamus in comparison with these in the LPS + M and LPS + H groups.

Our previous research found that gastric feeding could activate hypothalamic AMPK-autophagy and suppress POMC expression via gastrointestinal hormones, mainly via ghrelin, to ameliorate hypercatabolism compared with jejunal feeding [36]. However, in this study, serum and hypothalamic ghrelin in the endotoxemic rats did not increase with an increase in calories in EN. Ghrelin is an endogenous peptide,secreted mainly by gastric mucosal cells, that regulates appetite [53]. The regulatory factors for its secretion are still unknown. In rats with saline injected, the levels of ghrelin were nonsignificant among three EN groups. This suggests that the secretion of ghrelin may not be in a nutrition calorie dependent manner. Through the detection of the ileum, increased intestinal inflammatory factors and MUC2 expression and decreased ZO-1 level were observed with the an increase in calories in EN. LPS + H group showed only slightly benefit in protecting the intestinal barrier, the ZO-1 expression level in this group was not significantly different from that in LPS group. These suggested that endotoxemic rats may have a certain degree of intestinal dysfunction in the acute phase (3—4 days). During this period, appropriate EN helps in protecting intestinal function, whereas excessive EN may aggravate the intestinal load, thus aggravating intestinal inflammation and mucosal damage, and decreasing ghrelin secretion. Even if it has no effect on the long-term prognosis, it is not beneficial to the metabolic regulation of rats during this period. This finding is supported by that of a study by Chao Wu et al., who found that 20% EN provides the maximm protection to the intestinal mucosal barrier in rats with ischaemia reperfusion injury. More studies are needed to explore the optimal calories of EN required to regulate metabolism with disease progression and recovery of the intestinal function [24].

In addition, we also found EN could mitigate serum GLP-1 and PYY. And high calorie of EN showed weaker effect on serum PYY and PYY compared with LPS + L and LPS + M groups. Most effects of PYY and GLP-1 are inhibitory, including reductions in gastrointestinal motility, inhibition of gallbladder emptying, and inhibition of gastric, intestinal and pancreatic secretion, which leads to reduced digestion and absorption of nutrients.

Apart from ghrelin, the activation of the hypothalamic inflammatory system is also involved in the wasting of skeletal muscle in sepsis through the HPA axis [31]. In this study, we also measured the inflammatory factors in the serum to represent hypothalamic inflammation. We found that EN can reduce the inflammatory factor levels to some extent, however as nutrition increased, the inflammatory cytokine levels increased in the blood and in the intestine. In addition, we also found EN could mitigation serum GLP-1 and PYY. And high calorie of EN showed weaker effect on serum PYY and PYY compared with LPS + L and LPS + M groups. Most effects of PYY and GLP-1 are inhibitory, including reductions in gastrointestinal motility, inhibition of gallbladder emptying, and inhibition of gastric, intestinal and pancreatic secretion, which leads to reduced digestion and absorption of nutrients.

## Limitation

This experiment indicated only the metabolic regulatory effect of EN on the acute phase (the first three days) after LPS injection, and further research is required to determine the optimal amount of calorie at any time point in the chronic phase. In addition, in this experiment, we did not use the classic cecal ligation and puncture model of sepsis, because it presents many uncontrollable factors and has a large coefficient of variation for intestinal injury. Therefore, we only used a relatively controllable LPS injection model, although this model could not accurately simulate the pathophysiological response of sepsis. Therefore, more research is needed to investigate on the specific mechanism and influencing factors of EN involved in regulating metabolism.

## Conclusion

To the best of our knowledge, this study is the first to compare the effects of different calories of EN on the metabolic regulation of rats in the acute phase of endotoxemia. Compared with medium-calorie and high-calorie EN, the low-calorie EN effectively increased serum and hypothalamic ghrelin concentration and reduced inflammatory factors, which could suppress the expression of POMC to ameliorate hypercatabolism. Therefore, low-calorie EN may be more effective in regulating hypercatabolism cause by endotoxemia in the acute phase, and may be the preferred nutritional calorie in the early stage of endotoxemia. However, the optimal amount of calories for regulating the metabolism in the non-acute phase must be determined.

## Supplementary Information

**Additional file 1**. Chart S1. Experimental group design and sample size. NS: normal saline; LPS: Lipopolysaccharide; F: no feeding; L: 40kcal/kg/d EN; M: 80kcal/kg/d EN; H: 120kcal/kg/d EN.

## Data Availability

The datasets used and/or analysed during the current study are available from the corresponding author on reasonable request.

## Abbreviations

EN: Enteral nutrition
POMC: Proopiomelanocortin
ELISA: Enzyme-linked immunosorbent assay
LPS: Lipopolys-accharide
GHS-R1α: Growth hormone secretagogue receptor-1α
BW: Body weight
EDL: Extensor digitorum longus
GLP-1: Glucagon-like peptide-1
PYY: Pancreaticpolypeptide
AGRP: Agouti-related protein
CART: Cocaine-and amphetamine—regulated transcript
NPY: Neuropeptide Y
Akt: Protein kinase B
mTOR: Mammalian target of rapamycin
MAFBx: Muscle atrophy F-box
MURF-1: Muscle ring-finger-1
ZO-1: Zonula occludens 1
MUC2: Mucin-2
