# Supplementary figures and images for: Comparison of the effects of different calorie amounts of enteral nutrition in hypercatabolism associated with ghrelin-POMC in endotoxemic rats
Source: Nutr Metab (Lond). 2022 Apr 15;19:28. doi: 10.1186/s12986-022-00663-7 (PMC9013094; doi:10.1186/s12986-022-00663-7)

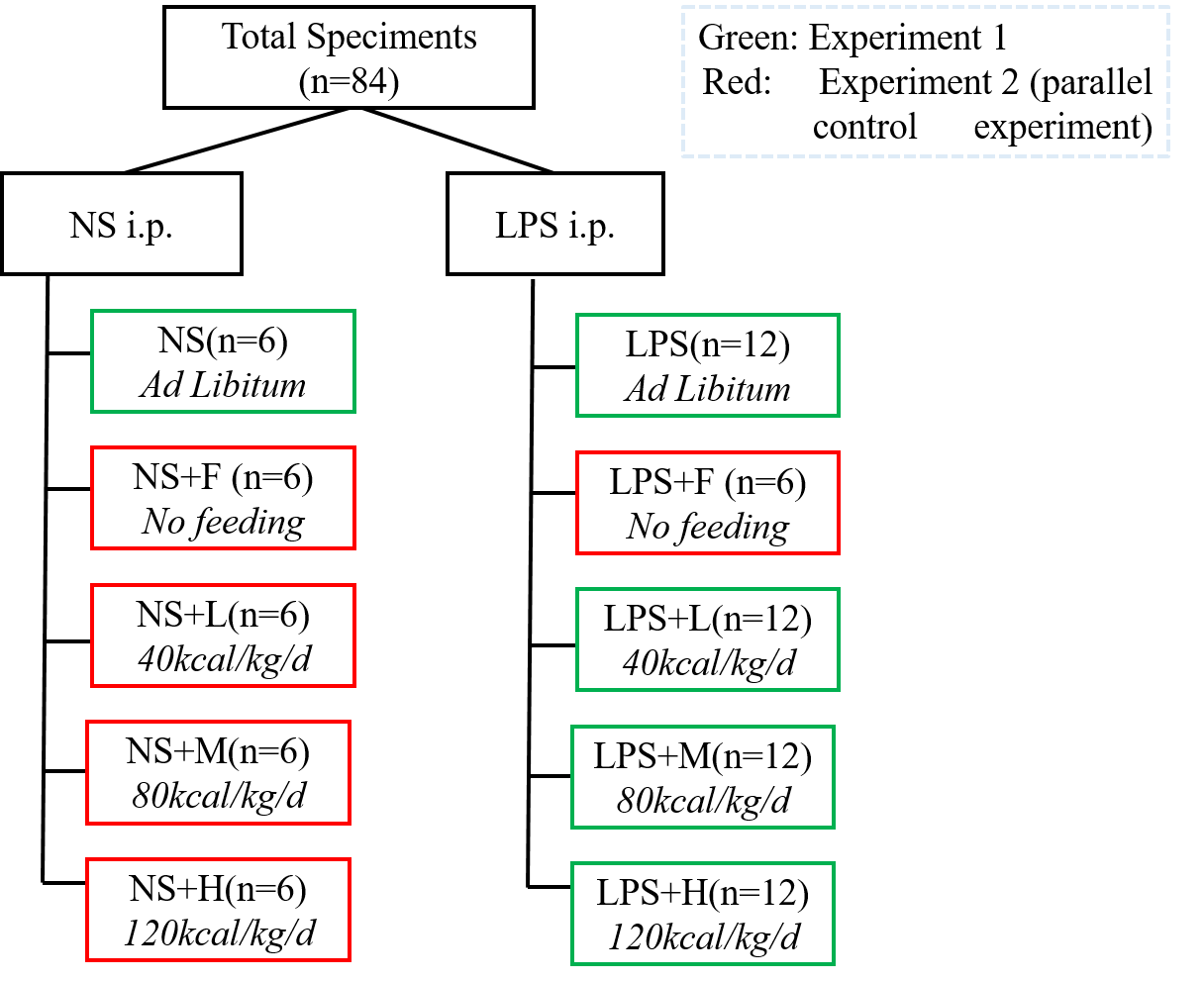

Supplement: Supplementary file 1 — Additional file 1. Chart S1. Experimental group design and sample size. NS: normal saline; LPS: Lipopolysaccharide; F: no feeding; L: 40kcal/kg/d EN; M: 80kcal/kg/d EN; H: 120kcal/kg/d EN. [file 12986_2022_663_MOESM1_ESM.tif]
